# GC-MS/MS analysis of metabolites derived from a single human blastocyst

**DOI:** 10.1007/s11306-021-01770-x

**Published:** 2021-01-25

**Authors:** Naomi Inoue, Yoshihiro Nishida, Emi Harada, Kumiko Sakai, Hisashi Narahara

**Affiliations:** Department of Obstetrics and Gynecology, Faculty of Medicine, Oita University, 1-1 Idaigaoka, Hasama-machi, Yufu, Oita, 879-5593 Japan

**Keywords:** Branched-chain amino acids, Culture medium, Embryo quality, Metabolomics

## Abstract

**Introduction:**

The field of assisted reproductive technology (ART) has significantly advanced; however, morphological evaluation remains as the chosen method of assessment of embryo quality.

**Objective:**

We aimed to examine metabolic changes in embryo culture medium to develop a non-invasive method for evaluation of embryo quality.

**Methods:**

We performed metabolic analysis of culture medium obtained from a single blastocyst cultured for freezing.

**Results:**

In total, 187 (39.8%) of the 469 detectable organic acid metabolites were identified. A significant change (*p* < 0.05) was observed in eight metabolites between the good-quality and poor-quality embryo groups. Differences were observed in several metabolic pathways between the good-quality and poor-quality embryo groups. Metabolites that showed significant changes were primarily involved in the metabolism of branched-chain amino acids.

**Conclusion:**

The quantification of metabolism in human embryos may assist in identification and selection of good-quality embryos with high rates of survival before freezing and implantation in conjunction with morphological classification. This may help to identify embryos with high rates of survival.

**Supplementary Information:**

The online version of this article (10.1007/s11306-021-01770-x) contains supplementary material, which is available to authorized users.

## Introduction

Since the birth of the first child through in vitro fertilisation in 1978 (Steptoe and Edwards 1978), the field of assisted reproductive technology (ART) has considerably evolved, and outcomes for children born through this procedure continue to improve. In vitro fertilisation techniques have developed in multiple ways, including preimplantation diagnosis as a means of evaluating the embryo (Penzias et al. 2018). Currently, the morphological evaluation of embryos based on the classifications described by either Veek (1999) or Gardner and Schoolcraft (1999) is primarily used to assess embryo quality. However, the morphological evaluation of embryos involves the subjective evaluation by each observer. Additionally, the morphology of the embryo does not necessarily reflect the successful implantation ability of the embryo. The number of studies examining non-invasive embryo evaluation methods, including the evaluation of genetic characteristics (Huang et al. 2019) and oxidation state (Alegre et al. 2019), has been increasing in recent years. To evaluate embryo quality, we analysed spent embryo culture medium for primary metabolites (organic acids, fatty acids and so on) absorbed or secreted by a single embryo. The purpose of this study was to perform exhaustive metabolomic analysis to subsequently analyse the differences in the metabolome between good- and poor-quality embryos and to evaluate the index of embryo development. Metabolic studies on embryos have been conducted using various animal species. One study providing a metabolic comparison of good- and poor-quality embryos in goats has been reported (Zhang et al. 2018); however, to the best of our knowledge, no study has simultaneously evaluated the morphological and exhaustive metabolic changes in a single human embryo using GC-MS/MS analysis. In this study, we aimed to examine the metabolic changes in culture medium obtained from a single blastocyst of each morphological classification, as determined by Gardner’s classification, to add a quantitative evaluation of human embryo quality.

## Materials and methods

### Collection of culture medium

Embryo culture medium samples were collected from embryos produced by conventional in vitro fertilisation (cIVF), between January and December 2018 from the Oita University Department of Obstetrics and Gynecology, after normal fertilisation was confirmed. Embryos had been cultured for long-term freezing. In this study, samples were classified according to their morphological characteristics; the means of follicle stimulation were not included in the analysis. Informed consent was obtained from all individual participants (all couples who provided samples) by a unified document in this study. The couples’ personal information was blinded, and samples were selected only for the morphological evaluation of the embryo. The study was approved by the Ethics Committee of Oita University Hospital (approval number 1317, Oita University).

### Sample preparation

After confirmation of successful fertilisation (day 1), each embryo was cultured for 6 days (day 6) to form the blastocyst. Embryos were cultivated individually in a single continuous culture containing 70 µl of broth medium. The samples were covered with oil to prevent evaporation of the nutrient solution. At the end of the culture, 50 µl of embryo culture medium was collected from each embryo and stored at  − 80 ℃.

The nutrient solution used was HiGROW OVIT (Fuso Pharmaceutical Industries Ltd.), with the addition of 10% Serum Substitute Supplement (Fuji Film Wako Pure Chemical Co., Ltd.). Embryos and cultures were added in 12- or 22-well plates (ART Culture dish, NIPRO) and cultured in a non-heating type dry incubator (EC65, ASTEC Inc.). Control samples included medium only and contained no embryos. Data on baseline characteristics of the female and male factors are shown in Supplementary 1.

### Morphological evaluation

Morphological evaluation was performed by expert embryologists using an inverted microscope (ECLIPSE, TE300, Nikon Co., Japan). On day 6, we classified the embryos into three quality groups; good-quality embryos were those which scored a 3BB or above based on the Gardner classification, poor-quality embryos were defective embryos which scored lower than 3BB, and embryos with developmental arrest were those with no visible growth (Table 1).

**Table 1:** Morphological data and day of classification of embryo samples used in this study

Group	Day of evaluation	Morphology
Good		
1	Day 5	3AB
2	Day 6	4AB
3	Day 5	3AA
Poor		
1	Day 6	4CC
2	Day 6	4CC
3	Day 6	4CC
Death		
1	Day 6	G4 3cell (arrest)
2	Day 6	G3 7cell (arrest)
3	Day 6	G4 3cell (arrest)

Based on the Gardner classification, 3BB is the threshold for good-quality embryos; below, 3BB is classified as poor-quality

### Measurements

Frozen embryo medium samples were thawed, phosphate-buffered saline was added to each 50 μl culture sample to obtain a total volume of 200 μl, and lipids were extracted using the method reported by Bligh and Dyer (1959). An internal standard, 5 μl of 2-isopropylmalic acid, was added, and centrifugation was performed for 7 min at 15,338 × *g*. The organic acids in the top aqueous layer were recovered into a new tube and dehydrated to form pellets. Then, 40 μl of methoxamine hydrochloride was added to facilitate the release of the pellets from the bottom of the tube by ultrasonic vibration. After shaking for 90 min at 30 ℃, 20 μl of N-methyl-N-trimethylsilyl trifluoroacetamide (MSTFA) was added, and the mixture was subjected to shaking for a further 30 min at 37 ℃. Following centrifugation, the supernatant was recovered and passed through a gas chromatography-mass spectrometer (GC-MS/MS-TQ8040; SHIMADZU, Inc., Japan) to identify primary metabolites.

### Statistical analyse

Statistical analysis was performed using multivariate analysis software (SIMCA13, Umetrics, Inc., Sweden). A *p*-value < 0.05 indicated statistical significance. Orthogonal partial least squares regression discriminant analysis (OPLS-DA) was used to cluster embryo quality groups and controls, and S-plot analysis was used to compare significant changes in metabolites between quality groups. Metabolic set enrichment analysis (MSEA) and metabolic pathway analysis (MetPA) were also used to confirm the significant pathways in the results of these measurements.

## Results

**Fig. 1:**
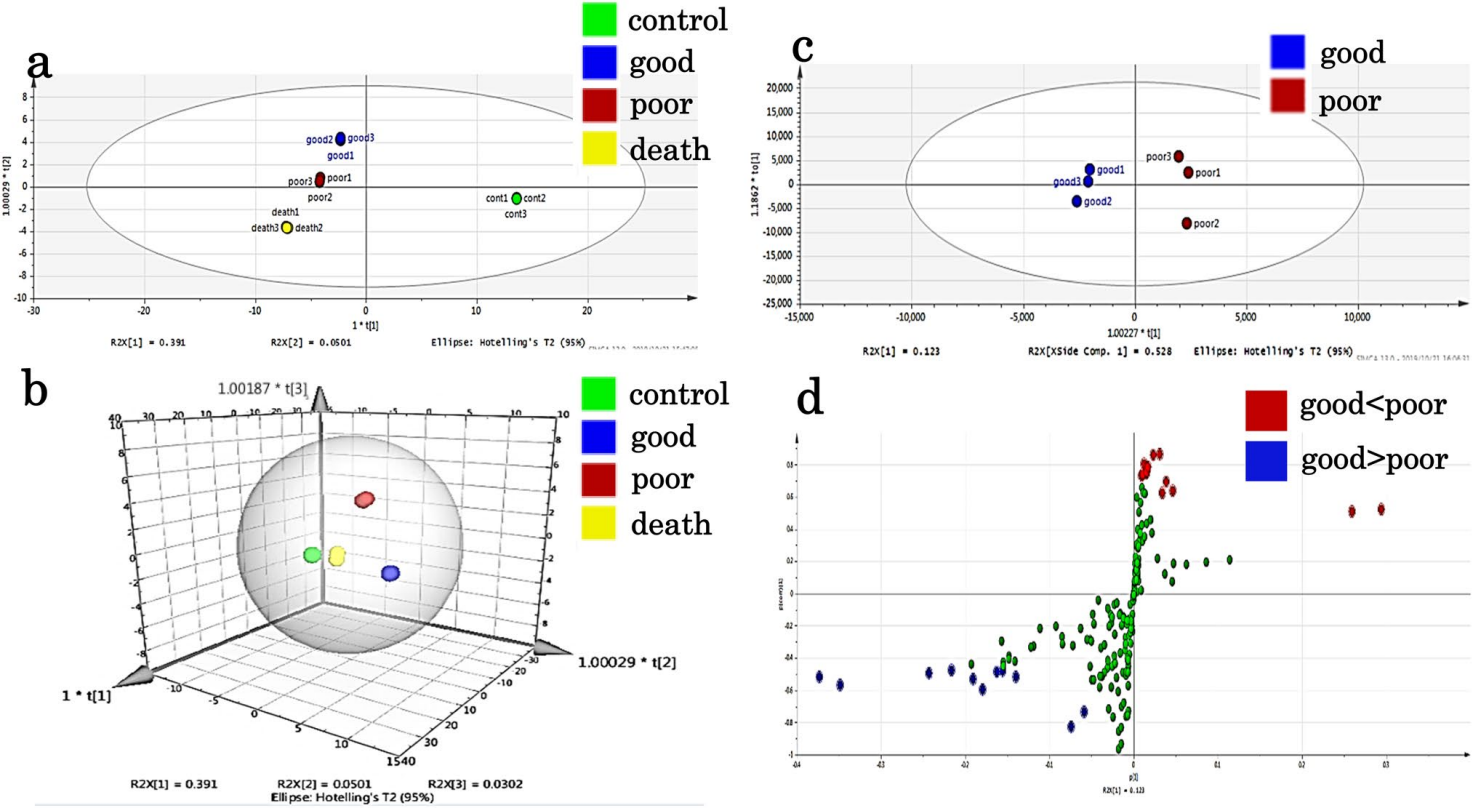
Results of the multiple classification analysis using SIMCA13, Umetrics, Inc., Sweden. **a** Distribution map of the four quality groups by OPLS-DA. All samples of each group show similar distribution. The x-axis t [1] and y-axis t [2] represent score vectors summarising all variables of the analysis. The goodness of prediction value R2 [1] = 0.391 and R2 [2] = 0.0501. The ellipse shows the 95% confidence interval using Hotelling’ T2 statistics. **b** 3D view of the four quality groups by OPLS-DA. The x-axis t [1], y-axis t [2], and z-axis t [3] represent score vectors summarising all variables of the analysis. The goodness of prediction value R2 [1] = 0.391, R2 [2] = 0.0501, and R2 [3] = 0.0302. The ellipse shows the 95% confidence interval using Hotelling’ T2 statistics. **c** Distribution map of the good-quality group vs poor-quality group by OPLS-DA. The distribution is remarkable between the good- and poor-quality groups. The x-axis t [1] and y-axis to [1] represent score vectors summarising all variables of the analysis. The goodness of prediction value R2 [1] = 0.123 and R2 to [1] = 0.528. The ellipse shows the 95% confidence interval using Hotelling’ T2 statistics. **d** S plot analysis of the good-quality group vs poor-quality group. OPLS-DA = orthogonal partial least square regression discriminant analysis. Positive or negative correlation levels are generally defined as  > 0.8 or  <  − 0.8, respectively

**Fig. 2:**
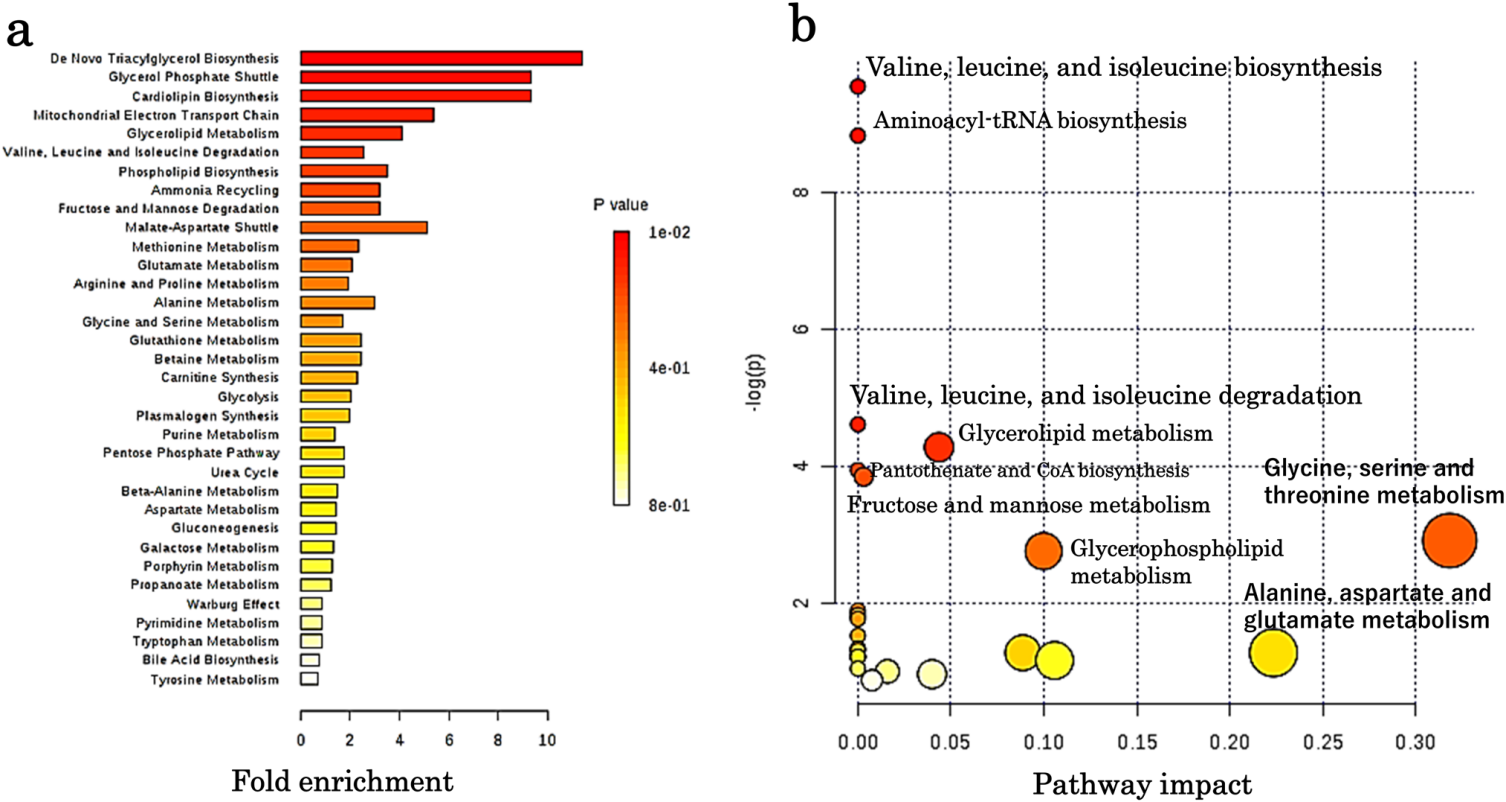
Results of pathway analysis using MetaboAnalyst. **a** Result of metabolic set enrichment analysis (MSEA) showing threefold enrichment of the valine, leucine, and isoleucine degradation pathways. **b** Result of metabolic pathway analysis (MetPA). Pathway impact value (x-axis) from pathway topology analysis and *p*-values from the pathway enrichment analysis (y-axis) are shown. MetaboAnalyst was developed by Dr. Jianguo Xia of the Institute of Parasitology at McGill University. It is a free software for metabolic analysis. (http://www.metaboanalyst.ca/feces/home.xhtml)

**Fig. 3:**
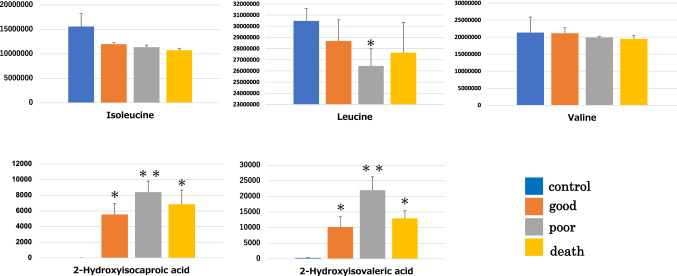
Variation in metabolomic changes between the poor-quality and good-quality embryo groups. Valine, leucine, and isoleucine levels are lower in the poor-quality group compared with those in the good-quality group. *p value < 0.05 compared with control. **p value < 0.01 compared with control

Of the 469 organic acid metabolites that could be analysed by metabolic analysis, 187 (39.8%) were identified. The metabolic profile of each embryo quality group, good-quality, poor-quality, developmental arrest (death), and control (medium only), was clearly identifiable by OPLS-DA, which showed a clear distinction between the control and test groups (Fig. 1a, b). The distribution of good- and poor-quality groups was also statistically distinguishable (Fig. 1c). S-plot analysis of the good- and poor-quality groups (Fig. 1d) showed a difference in the level of metabolites between the two groups since the point was far from the center; 13 metabolites showed increased levels, while 11 showed decreased levels in the poor-quality groups compared with good-quality groups. Additionally, six of the one hundred eighty metabolites were significantly increased (*p* < 0.05) while two were significantly reduced (*p* < 0.05) in the poor-quality group compared to those in the good-quality group (Table 2). The metabolic pathways identified as significant, using the Metabolite Set Enrichment Analysis (MSEA) and Metabolomic Pathway Analysis (MetPA), were involved in mitochondrial and amino-acid metabolism (Fig. 2a, b). Thus, we considered that the poor-quality group produced more energy than the good-quality group. Interestingly, both 2-hydroxyisovaleric acid and 2-hydroxyisocaproic acid levels were high in the poor-quality group and not included in culture media intrinsically. They are metabolites from valine, leucine, and isoleucine defined as branched-chain amino acids. Although valine, leucine, and isoleucine levels were lower in the poor-quality group, no significant difference was observed between the two groups (Fig. 3).

**Table 2:** Organic acids evaluated by S plot analysis

(a)	Good		Poor		*p* value
	Average	SD(±)	Average	SD(±)	
2-Hydroxyisovaleric acid	10,208.67	3265.685	21,972.67	4317.334	0.018586
3-Methylcrotonoylglycine	3788.333	739.4964	5858.667	810.7183	0.027955
Dihydroxyacetone phosphate	7125.667	1164.786	10,405.33	1414.169	0.032277
Glycerol 3-phosphate	19,412.33	852.4828	22,957.67	1831.116	0.034021
5-Methoxytryptamine	4254.333	311.1498	7999	2303.596	0.042477
2-Hydroxyisocaproic acid	9539.333	1782.132	13,382.33	1780.663	0.048592
Pimelic acid	3126.333	1064.42	4731.333	134.2543	0.050918
Citramalic acid	3274	844.2859	4516.667	328.6846	0.062209
3-Phenyllactic acid	58,816.33	16,009.84	81,883.33	7794.977	0.070451
Galactitol	20,098	7013.909	58,162.33	29,930.47	0.077409
O-Phosphoethanolamine	122,448.7	10,774.51	142,411	14,566.94	0.097103
Dimethylglycine	4079	1268.637	5470.667	499.4546	0.11118
3-Hydroxyglutaric acid	6,659,492	1,660,659	8,071,087	568,138.8	0.159442

(b)	Good		Poor		*p* value
	Average	SD(±)	Average	SD(±)	
Uridine	154,391.3	33,253.43	80,985	10,369.91	0.020363
Tryptamine	156,819.3	31,962.06	104,001	8490.839	0.043403
Isoleucine	11,970,899	325,692.7	11,351,353	449,953.2	0.094921
Hydroxylamine	7,194,791	443,351.5	6,743,480	180,671.4	0.126671
Leucine	28,682,819	1,896,338	26,448,798	1,565,656	0.134116
Glycine	40,635,422	601,859.1	37,613,723	3,416,155	0.142719
Mannitol	8,486,584	609,098	7,751,260	601,632.9	0.145648
Allose	7,892,075	393,262.1	7,305,359	589,071.1	0.153211
Mannose	6,228,571	342,985.6	5,614,818	668,722.7	0.156211
Aspartic acid	22,350,676	501,649.8	21,218,557	1,305,195	0.158028
Valine	21,125,490	1,643,932	19,884,846	287,107.8	0.176205

**a** Organic acid levels increased in the poor-quality group compared to those in the good-quality group. **b** Organic acid levels decreased in the poor-quality group compared to those in the good-quality group

## Discussion

In this study, we comprehensively analysed metabolites of culture medium from a single blastocyst. Differences were observed in a few metabolic pathways in the poor-quality group compared with those in the good-quality group. We focused on amino-acid metabolism specifically. Amino acids are necessary for the development of embryos. The importance of amino acids in embryo culture medium has been reported by Brinstar in the mid-1960s (Brinster 1965). Thereafter, the requirement of amino acids for embryo development has been studied in various animal species including mice (Chatot et al. 1989; Ho et al. 1995; Nakazawa et al. 1997; Lane and Gardner 1998), rats (Miyoshi et al. 1995), sheep (Gardner et al. 1994), bovine (Rosenkrans et al. 1994; Lee and Fukui 1996), and pigs (Petters et al. 1990; Yoshioka et al. 2002). Several studies have reported that amino acids promote the differentiation of embryos in humans (Devreker et al. 2001; Winkle 2001; Houghton et al. 2002). Houghton et al. and Devreker et al. have reported that amino-acid metabolism is enhanced in embryos with arrested development compared to that in well-developing embryos. In the present study, it was observed that the morphological classification of embryos was correlated with amino-acid metabolism, and specifically that the metabolic pathways of valine, leucine, and isoleucine were enhanced in poor-quality embryos. Our observation is consistent with that of previous studies reporting that metabolism is enhanced in poorly developing embryos. Metaboanalyst showed that valine, leucine, and isoleucine biosynthesis / degradation were concerned in differences of metabolites of poor- and good quality groups. Essential amino-acid metabolism as implied via valine, leucine, and isoleucine biosynthesis/degradation was linked to the TCA cycle and was related to energy production (Supplementary 2). The metabolic pathways involved in mitochondrial metabolism were also enhanced in the poor-quality group. The poor-quality group requires more energy for the maintenance of cells than the good-quality group.

Bracewell-Milnes et al. (2017) have suggested a non-invasive embryo assessment to improve IVF outcome using spent culture medium, while others have suggested Raman, NMR, or NIR spectroscopy, and HPLC-MS. Uyar and Seli (2014) have indicated that GC-MS is a cumbersome procedure with long analysis time. Our GC-MS / MS technique required only a short period for analysis; therefore, there is a sufficient time to choose the best embryo suitable for transfer; hence we suggest that this method will be useful for assessment of the quality of embryos.

There were a few limitations of this study. The sample size was limited, and it was not possible to evaluate reproducibility using the same samples because all the available samples were used. In this study, the method of IVF was limited to cIVF, and different results might be obtained had the same study been carried out with ICSI. The study also did not examine pregnancy and labour; the establishment of pregnancy and the result of labour are determined not only by the morphological evaluation of the embryo but also by the involvement of various factors such as the intrauterine environment and maternal conditions. It is possible that a correlation between metabolic changes and pregnancy may be observed.

In this study, we analysed metabolites in the culture media derived from a single human embryo. Statistical analyses revealed that there were differences in metabolic pathways between poor- and good-quality embryos. This metabolome-based technique may thus be useful for the non-invasive and specific evaluation of embryo quality, in which changes in metabolites may be correlated with morphological classifications to select good-quality embryos. By performing this analysis prior to freezing the embryos, it may be possible to select embryos with a higher survival rate. However, it would be necessary to clarify the relationship between metabolites and pregnancy outcomes. We anticipate that the clinical application of our method may be used with morphological evaluation in the future.

## Electronic supplementary material

Below is the link to the electronic supplementary material.

Supplementary file1 (PPTX 49 kb)

## Data Availability

The datasets generated during and/or analysed during the current study are available from the EMBL-EBI MetaboLights database (https://doi.org/10.1093/nar/gkz1019, PMID: 31691833) with the identifier MTBLS2189.
