# Supplementary material for: GC-MS/MS analysis of metabolites derived from a single human blastocyst
Source: Metabolomics. 2021 Jan 25;17(2):17. doi: 10.1007/s11306-021-01770-x (PMC7835178; doi:10.1007/s11306-021-01770-x)
Supplement: Supplementary file 1 — Supplementary file1 (PPTX 49 kb) [file 11306_2021_1770_MOESM1_ESM.pptx]

## Slide 1
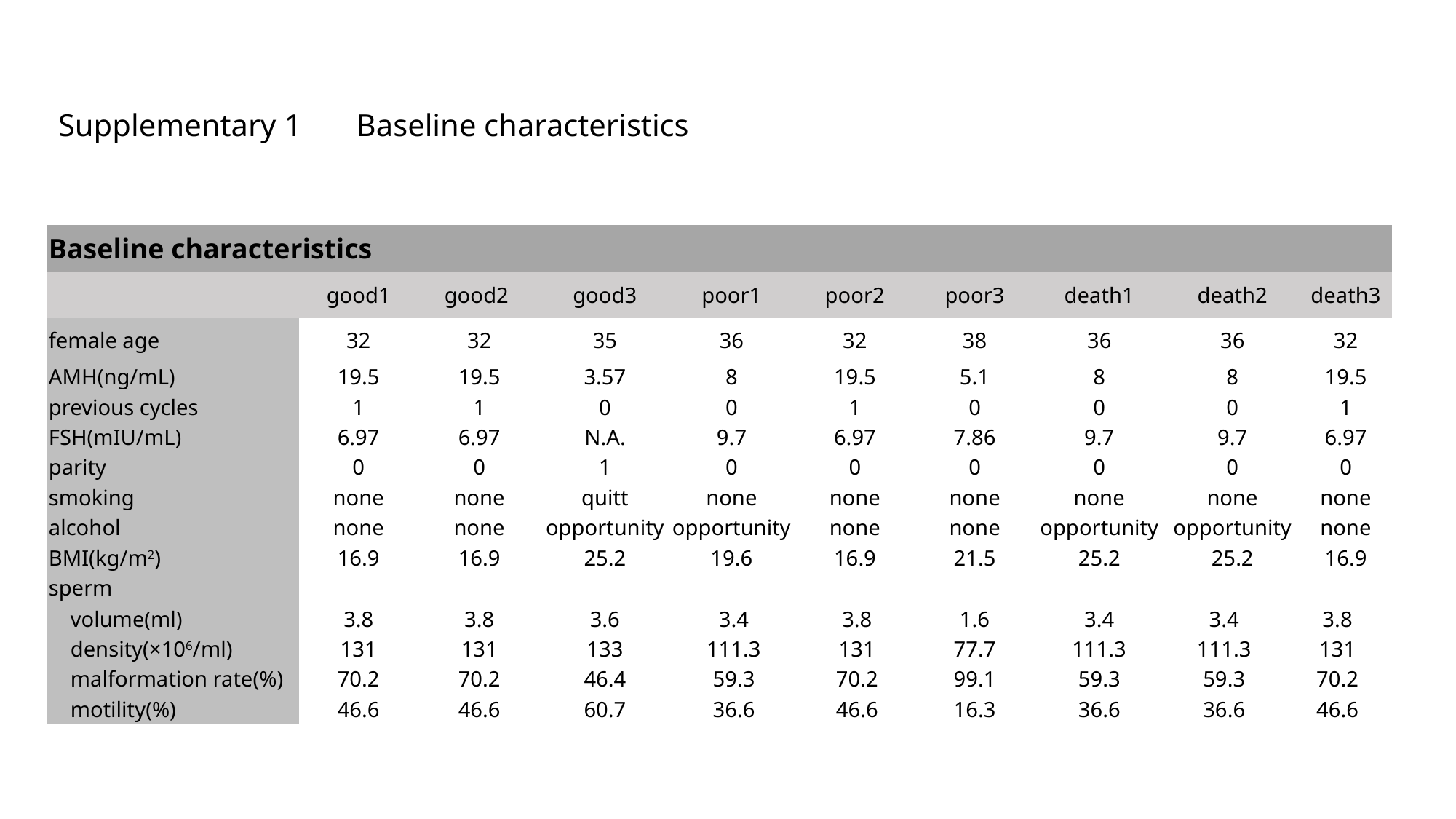

Supplementary 1 Baseline characteristics
| Baseline characteristics | | | | | | | | | | | |
| --- | --- | --- | --- | --- | --- | --- | --- | --- | --- | --- | --- |
| | good1 | good2 | good3 | poor1 | poor2 | poor2 | poor3 | death1 | death2 | | death3 |
| female age | 32 | 32 | 35 | 36 | 32 | 32 | 38 | 36 | 36 | | 32 |
| AMH(ng/mL) | 19.5 | 19.5 | 3.57 | 8 | 19.5 | 19.5 | 5.1 | 8 | 8 | | 19.5 |
| previous cycles | 1 | 1 | 0 | 0 | 1 | 1 | 0 | 0 | 0 | | 1 |
| FSH(mIU/mL) | 6.97 | 6.97 | N.A. | 9.7 | 6.97 | 6.97 | 7.86 | 9.7 | 9.7 | | 6.97 |
| parity | 0 | 0 | 1 | 0 | 0 | 0 | 0 | 0 | 0 | | 0 |
| smoking | none | none | quitt | none | none | none | none | none | none | | none |
| alcohol | none | none | opportunity | opportunity | none | none | none | opportunity | opportunity | | none |
| BMI(kg/m2) | 16.9 | 16.9 | 25.2 | 19.6 | 16.9 | 16.9 | 21.5 | 25.2 | 25.2 | | 16.9 |
| sperm | | | | | | | | | | | |
| volume(ml) | 3.8 | 3.8 | 3.6 | 3.4 | | 3.8 | 1.6 | 3.4 | 3.4 | 3.8 | |
| density(×106/ml) | 131 | 131 | 133 | 111.3 | | 131 | 77.7 | 111.3 | 111.3 | 131 | |
| malformation rate(%) | 70.2 | 70.2 | 46.4 | 59.3 | | 70.2 | 99.1 | 59.3 | 59.3 | 70.2 | |
| motility(%) | 46.6 | 46.6 | 60.7 | 36.6 | | 46.6 | 16.3 | 36.6 | 36.6 | 46.6 | |

## Slide 2
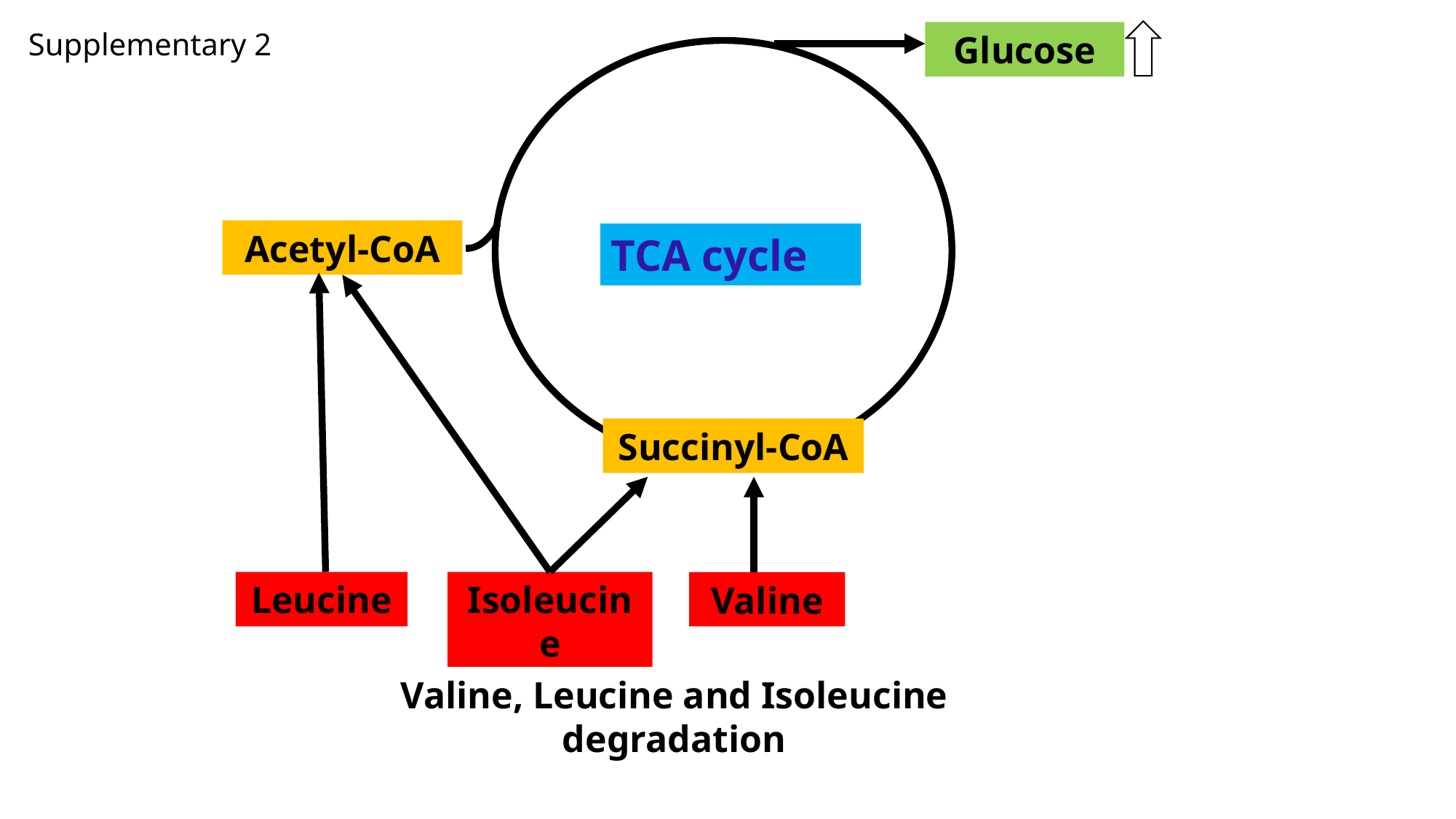

Supplementary 2
Glucose
Acetyl-CoA
TCA cycle
Succinyl-CoA
Leucine
Isoleucine
Valine
Valine, Leucine and Isoleucine degradation
